# Dysphagia and masticatory performance as a mediator of the xerostomia to quality of life relation in the older population

**DOI:** 10.1186/s12877-020-01901-4

**Published:** 2020-12-02

**Authors:** Ting-Yu Lu, Jen-Hao Chen, Je-Kang Du, Ying-Chun Lin, Pei-Shan Ho, Chien-Hung Lee, Chih-Yang Hu, Hsiao-Ling Huang

**Affiliations:** 1 Department of Oral Hygiene, College of Dental Medicine, Kaohsiung Medical University, No. 100 Shih-Chuan 1st Road, Kaohsiung City, 807 Taiwan; 2 Department of Oral Hygiene, Hsin Sheng Junior College of Medical Care and Management, Taoyuan City, Taiwan; 3 School of Dentistry, College of Dental Medicine, Kaohsiung Medical University, Kaohsiung City, Taiwan; 4 Department of Dentistry, Kaohsiung Medical University Hospital, Kaohsiung City, Taiwan; 5 Division of Medical Statistics and Bioinformatics, Department of Medical Research, Kaohsiung Medical University Hospital, Kaohsiung Medical University, Kaohsiung City, Taiwan; 6 Department of Public Health, College of Health Sciences and Research Center for Environmental Medicine, Kaohsiung Medical University, Kaohsiung City, Taiwan; 7 School of Public Health, Health Sciences Center, Louisiana State University, New Orleans, LA USA

**Keywords:** Xerostomia, Dysphagia, Masticatory performance, Oral health-related quality of life, Path analysis

## Abstract

**Background:**

The impact of poor oral health on older adults’ quality of life is a public health problem. In this study, the mediating effects of dental status, occlusal condition, dysphagia, and masticatory performance on the association between xerostomia and oral health-related quality of life (OHRQoL) were assessed in the older adult population.

**Methods:**

Stratified cluster sampling was used to recruit 1076 community-dwelling adults aged 65 years and older from Kaohsiung, Taiwan. Community care centers were randomly selected according to their geographic classifications (urban, rural, or mountainous areas). Assessments of dental status and occlusal condition were performed by dentists. Information on demographics, physical function, xerostomia, dysphagia and depression was collected through face-to-face interviews. Masticatory performance was evaluated using color-changeable chewing gum. OHRQoL was measured using the Geriatric Oral Health Assessment Index. Hierarchical regression models were used to assess the relationships between OHRQoL and physical function, dental status and oral function in older adults. Path analysis was used to estimate direct and indirect pathways between xerostomia and OHRQoL.

**Results:**

Participants with xerostomia exhibited a 0.20 OHRQoL reduction (*p* < .001) compared with patients with no xerostomia, and the direct effect accounted for 83.3% of the total effect. Dysphagia and masticatory performance were found to exert significant mediating effects on the association between xerostomia and OHRQoL (βs = 0.20 and − 0.12, respectively; both *p* < .001; βs = 0.06 and − 0.09, respectively; both *p* < .05). Moreover, potential mediating effects of the number of functional teeth (βs = − 0.11 and − 0.43, respectively; both *p* < .001) and occlusal condition (βs = 0.09 and 0.13, respectively; both *p* < .05) on the relationship between xerostomia and masticatory performance were noted.

**Conclusions:**

Dysphagia and masticatory performance may serve as pathways through which xerostomia affects quality of life. Early oral function intervention may be a valuable and actionable target for older adults to maintain quality of life. Our results further suggest that checkup and screening for oral dysfunction are essential to prevent or delay the onset of complications.

## Background

Xerostomia is defined as subjective dryness of the mouth, which can occur during normal aging. According to relevant data published in 2018, the estimated prevalence of dry mouth ranges from 1 to 62% [1], with a higher prevalence among older people. One study reported greater crown surface decay in individuals with xerostomia than in those without xerostomia and proposed that decayed crown surfaces may increase the risk of dental caries and cause tooth loss, with missing teeth shown to be associated with subjective sensation of dry mouth [2]. Xerostomia may also affect speech, chewing, swallowing, denture wearing, and oral health-related quality of life (OHRQoL) [3], as well as further impede activities of daily living [4]. Overall, xerostomia is considered a serious oral health complaint, and it has numerous effects on oral health, which may in turn negatively affect quality of life.

Normal swallowing comprises three phases: oral, pharyngeal, and esophageal phases. In the oral phase, food is chewed and mixed with saliva to form a bolus, which can then be passed to the oropharynx (the upper part of the throat) and prepared for swallowing. Bolus formation during chewing requires saliva, which is therefore crucial in the oral phase [5]. Swallowing dysfunction is common among older adults, and its prevalence increases with age and frailty. Dysphagia is defined as any disruption to the swallowing process. Physiological and functional changes in the swallowing process due to aging are referred to as presbyphagia. Neurogenic dysphagia is dysphagia associated with neurological conditions such as dementia, Parkinson’s disease, or stroke [5]. Moreover, physiological changes resulting from diseases such as head and neck cancer, cervical spine surgery, or head injuries also lead to dysphagia [6].

Masticatory performance may affect the diets of older adults, including their nutrition intake. If tooth loss is also present, food comsumption may become difficult. Loss of masticatory function is also associated with increased disability and mortality. Improvements in masticatory function can enhance quality of life. A study in Japan showed that the prevalence of dissatisfaction with daily life was 3.4 times higher in individuals able to chew only ≤4 types of food than in those who could chew 15 types; dissatisfaction was 2.1 times greater in those who had 10–19 teeth than in those with ≥20 teeth [7]. Therefore, in older adults, improved chewing ability may increase satisfaction with daily living.

Studies have examined the relationships between dysphagia, oral health, masticatory performance and activities of daily living [8]. Quality of life was found to be lower in patients with dysphagia [9]. Older adults with subjective oral dryness are more likely to experience problems with chewing one or more types of foods and with activities of daily life, such as eating or speaking [2]. Although oral health has been reported to be associated with poorer quality of life [10, 11], whether other pathways influence perceived quality of life should be investigated. No study has investigated mediators between xerostomia and OHRQoL or explored possible pathways in oral function variables. Through an understanding of the effects of mediators, key elements can be identified and costs can be reduced to increase the efficacy of early intervention. Research has identified relationships of dental status and oral function with OHRQoL through multiple regression analysis [12, 13]. Path analysis is a specific structural equation modelling (SEM) tool that can replace multiple separate regressions to facilitate the examination of mediating effects within a single model [14]. It also enables the testing of causal relationships among a set of observed variables and provides a powerful tool for identifying factors mediating the association between exogenous variables and outcomes. This study employed path analysis to examine the pathway from xerostomia to OHRQoL with repect to some mediators in a comprehensive model for older people. We hypothesized that xerostomia is related to OHRQoL through the mediators of dental status, dysphagia, and masticatory performance.

## Methods

A cross-sectional study was conducted involving community-dwelling participants aged 65 years or older in Kaohsiung, Taiwan from May 2018 to January 2019. The study adopted a multistage, stratified cluster sampling method to select individuals from the older adults population. Kaohsiung City has 39 districts in three geographic classifications (urban, rural, and mountainous areas). In the first stage of sampling, 25 districts were randomly selected from the 39 districts according to the probability proportional to size sampling method. In the second stage, community care centers in the 25 districts were randomly selected. In the final stage, older adults aged 65 years or older were recruited from each community care center selected. In total, 1180 participants were recruited from urban areas (*n* = 609), rural areas (*n* = 555), and mountainous areas (*n* = 16).

Participants were excluded if they self-reported any of the following: (a) mental disorders, (b) expressive language disorders, (c) moderate or severe cognitive impairment, as determined by a short portable mental status questionnaire (SPMSQ) [15], (d) high-to-total dependence in Activities of Daily Living (ADL) [16], or (e) moderate-to-severe depression, as assessed using the Geriatric Depression Scale (GDS) [17]. The final analysis included 1076 participants (response rate: 91.2%).

According to the sample size criteria for SEM analysis, a large sample size was required for our highly complex model with numerous free parameters. The ideal sample size is generally considered to be 20 participants per free parameter [18]. This study had 11 free parameters, and 220 was therefore set as the minimum sample size.

### Instrument

A structured questionnaire was developed to collect data on demographics (i.e., age, sex, education level), depression level, oral function (i.e., xerostomia and dysphagia), physical function (i.e., frailty, sarcopenia), and OHRQoL. All instruments were translated from English to Chinese and back-translated to English by bilingual research staff and then verified for accuracy by two senior researchers. Items were reviewed by a panel of experts to assess content validity. The content validity index was 0.89–1.00. To ensure that the study participants understood the content, the questionnaires were pilot tested on 30 older adults. The reliability of each scale was assessed in terms of internal consistency (Cronbach’s alpha coefficient). Masticatory performance was assessed using color-changeable chewing gum (Xylitol, 3.0 g; Lotte, Saitama, Japan).

#### Dental examination

Dental examinations were performed by seven dentists according to World Health Organization criteria. The kappa coefficient of tooth decay was 0.77 for interrater agreement. The Kendall’s W coefficient for the plaque index was 0.87 for interrater agreement. The dental status and oral hygiene (i.e. plaque index and tongue coating) were recorded.

#### Oral health-related quality of life

OHRQoL was measured using the Geriatric Oral Health Assessment Index (GOHAI), which is a 12-item instrument with three domains: physical function (eating, speech, and swallowing), psychosocial function (worry or concern regarding oral health, dissatisfaction with appearance, self-consciousness regarding oral health, and avoidance of social interaction because of oral problems), and pain or discomfort (medication use to relieve oral pain or discomfort). The English version of the GOHAI was translated into Chinese for the participants (GOHAI-T) [19]. Each item in the GOHAI was rated on a 5-point Likert scale ranging from, 1 (*always*) to 5 (*never*). The total score ranged from 12 to 60 points, with a higher score indicating a more favorable OHRQoL. The Cronbach’s alpha indicated an internal consistency of 0.75 for the scales.

#### Xerostomia

A condenced version of the Xerostomia Inventory was used to identify and classify mouth dryness. Participants responded to five items: (a) “My mouth feels dry when I eat a meal,” (b) “My mouth feels dry,” (c) “I have difficulty eating dry foods,” (d) “I have difficulty swallowing certain foods,” and (e) “My lips feel dry.” Each item was assigned a score of 1 (*never*), 2 (*occasionally*), or 3 (*often*) [20]. The total score ranged from 5 to 15 points, with a higher score reflecting a higher level of mouth dryness. Participants with total scores of ≥10 points represented the top 50% of total xerostomia scores [21]. The Cronbach’s alpha indicated an internal consistency of 0.88 for the scale.

#### Dysphagia

The dysphagia variable was measured using 15 questions in the Ohkuma questionnaire to rapidly screen the community members; examples of questions included “Do you ever have difficulty swallowing?” “Do you ever have difficulty as a result of cough up phlegm during or after a meal?” “Does it take you longer to eat a meal than it used to?” “Do you feel that it is becoming difficult to eat solid foods?” and “Do you ever have difficulty sleeping because of coughing during the night?” [22]. Possible responses were “obviously” (*frequently*), “slightly” (*sometimes*), or “no” (*never*). Respondents with at least one severe symptom were classified as having dysphagia. The Cronbach’s alpha value was 0.85, indicating satisfactory internal consistency.

#### Masticatory performance

Masticatory performance was evaluated using color-changeable chewing gum. The gum changes to red when chewed because the yellow and blue dyes seep into saliva, and citric acid elution produces the red color [23]. Participants were asked to chew for 2 min. The observer assessed the color of the gum by using a color chart with five color gradations ranging from 1 (*very good*) to 5 (*very poor*). The masticatory performance scoring was simplified into three categories, 1–3 = *good*, 4 = *moderate*, 5 = *poor*.

#### Occlusal condition

Occlusal condition was measured using the Eichner index [24], which is based on the number of posterior occlusal contacts of functional teeth in the premolar and molar regions—called support zones. The posterior regions are divided into four support zones on both sides and are classified in Eichner categories A, B, or C. Class A has contacts in four support zones; class B has contacts in one to three support zones or only in the anterior region; and class C has no contact with any support zones, although a few teeth may remain.

#### Dental status

The variables related to dental status were the numbers of remaining natural teeth, functional teeth, fixed artificial teeth, complete dentures, and removable partial dentures. The number of functional teeth was defined as the number of natural teeth, excluding teeth with grade III mobility and residual roots, and fixed artificial teeth (i.e., abutment teeth, bridge, and implant-supported prostheses).

#### Oral hygiene status

The plaque index was used to record plaque scores for four surfaces (buccal, lingual, mesial, and distal) of tooth numbers 12, 16, 24, 32, 36, and 44; scores ranged from 0 to 3. Tongue coating was assessed using the Winkel tongue coating index [25]. The tongue was divided into six areas (three posterior and three anterior), and the coating was scored as 0 = *no coating*, 1 = *light coating*, or 2 = *severe coating*; scores ranged from 0 to 12 points.

#### Covariates

Physiological and psychologic or psychiatric state may potentially be affected by oral health status. Therefore, we examined age, sex, educational level, frailty and sarcopenia as the covariates. Moreover, because a self-reported data collection process was adopted, participants with cognitive impairment (moderate or severe cognitive impairment) were excluded from the study. Frailty was assessed using the Study of Osteoporotic Fractures index (SOF) [26]. Sarcopenia was assessed using the SARC-F self-administered questionnaire [27]. Finally, all covirates collected during the study were retained in the final model for pathway analysis.

### Data collection

Data were collected by thoroughly trained interviewers during face-to-face interviews in accordance with standard protocol. To prevent information bias during interviews, each interviewer attended a 1-h training course on the standard process and data collection criteria. The data collection process comprised three steps. First, a dentist performed a dental examination, and a dental hygienist recorded the dental status, plaque index score, and tongue coating score. Second, a structured questionnaire was administered by an interviewer in Mandarin or Taiwanese. The entire interview process took approximately 30–45 min. Finally, the research staff collected data on masticatory performance, assessed according to the results of participants chewing color-changing gum, and on physical function and frailty.

### Statistical analysis

Stata 13.1 (StataCorp LP, College Station, TX, USA) was used for statistical analysis. Participants were divided into two groups according to their xerostomia status. The demographic characteristics, dental status, oral function, and physical function were compared between the two groups by using chi-square tests and two-sample *t* tests. A multiple linear regression model was adjusted using the hierarchical method to obtain a predictive model for OHRQoL. To adjust the final model, changes in the adjusted R^2^ and F values were considered for each new independent variable added, and the variance inflation factor (VIF) was used to assess multicollinearity among all independent variables. Path analysis was then used to test a model exploring the relationships of xerostomia, dental status, oral function with OHRQoL and to identify both direct and indirect relationships in the model. Path analysis is specific to SEM and invloves the simultaneous analysis of the assumed relationships in multivariate data. SEM is one of the most favored statistical techniques in the social sciences and has become popular in dental science [28, 29]. In the present study, the following criteria were used to define model goodness of model fit: χ^2^/df < 3.00, root mean square error of approximation (RMSEA) < 0.08, and comparative fit index (CFI) ≥ 0.90. To obtain an acceptable model, initial fit, modification (using the motivational interviewing method in Stata), and rafting steps were followed [30]. After the fit of the full model was estimated, nonsignificant direct paths were removed to generate a statistically parsimonious model, after which reestimation was performed for comparison with the full model using a chi-square test. Finally, all the steps were repeated until a good fit was achieved for the model. Significance was set at *p* < .05 for all statistical tests.

## Results

Table 1 summarizes the characteristics of participants categorized according to xerostomia status. In total, 28.6% were men (mean age of 74.0 ± 6.5 years) and 71.4% were women (mean age of 74.0 ± 6.4 years). A sigfinicantly higher percentage of participants in the xerostomia group reported fewer than 20 natural teeth and functional teeth (59.6 and 54.6%, respectively) compared with those in the xerostomia-free group (39.6 and 34.9%, respectivley). Furthermore, a sigifnicantly higher percentage of participants in the xerostomia group exhibited class B and class C occlusal condition (47.1 and 28.6%, respectivley) compared with in the xerostomia-free group (37.5 and 19.0%, respectivley). The xerostomia group displayed poorer masticatory performance and more difficulty swallowing than the xerostomia-free group did (*p* < .001). Of the older people with xerostomia, 38.7 and 3.4% had prefrailty and frailty, respectively, compared with 16.6 and 1.0% in the xerostomia-free group (*p* < .001). The total GOHAI scores were 53.7 ± 6.3 in the xerostomia-free group and 47.9 ± 8.8 in the xerostomia group (*p* < .001).

**Table 1:** Basic information according to xerostomia group (*n*=1,076)

Variables	Total		Xerostomia-free (*n*=957)		Xerostomia (*n*=119)		*P*
	n	%	n	%	n	%	
Age (M±SD)^†^	74.0±6.4		73.7±6.3		76.5±6.6		<.001
Sex							0.03
Men	308	28.6	284	29.7	24	20.2	
Women	768	71.4	673	70.3	95	79.8	
Education level							<.001
Illiterate/ Elementary school	495	46.0	422	44.1	73	61.3	
Junior high/ High school	366	34.0	333	34.8	33	27.7	
Above technical school/ College	215	20.0	202	21.1	13	10.9	
BMI (M±SD)^†^	24.3±3.5		24.3±3.5		24.2±3.8		0.888
Number of natural teeth							<.001
< 20 teeth	450	41.8	379	39.6	71	59.6	
≥ 20 teeth	626	58.2	578	60.4	48	40.3	
Number of functional teeth							<.001
< 20 teeth	399	37.1	334	34.9	65	54.6	
≥ 20 teeth	677	62.9	623	65.1	54	45.4	
Occlusal condition							<.001
A	445	41.4	416	43.5	29	24.4	
B	415	38.6	359	37.5	56	47.1	
C	216	20.1	182	19.0	34	28.6	
Implant	45	4.2	43	4.5	2	1.7	0.148
Edentulous	94	8.7	79	8.3	15	12.6	0.113
Removable denture							
CD	202	18.8	171	17.9	31	26.1	0.031
RPD	319	29.7	269	28.1	50	42.0	0.002
PI (0- 3) (M±SD)^†^	0.9±0.5		0.9±0.5		1.0±0.7		0.569
Tongue coating (0-12) (M±SD)^†^	4.5±3.3		4.5±3.3		3.9±3.3		0.058
Masticatory performance							<.001
Good	373	35.1	350	37.0	23	19.5	
Moderate	410	38.6	364	38.5	46	39.0	
Poor	280	26.3	231	24.4	49	41.5	
Dysphagia	136	12.6	97	10.1	39	32.8	<.001
Sarcopenia	46	4.3	32	3.3	14	11.8	<.001
Frailty							<.001
Robust	857	79.7	788	82.3	69	58.0	
Pre-frailty	205	19.1	159	16.6	46	38.7	
Frailty	14	1.3	10	1.0	4	3.4	
GOHAI (M±SD) (0-60) ^†^	53.0±6.8		53.7±6.3		47.9±8.8		<.001

chi-square test; †two sample t test

*BMI* Body mass index, *CD* Complete denture, *RPD* Removable complete denture, *PI* Plaque index

Table 2 displays the results of the hierarchical linear regression analysis for the relationships of physical function, dental status, and oral function with OHRQoL level after adjustment for other independent variables. No multicollinearity was identified among the independent variables. Demographic factors, frailty, and sarcopenia were entered in the first step and explain 5.57% of the variance in OHRQoL, as measured by the total GOHAI scores (Model 1, Table 3). The additional variables related to dental status (i.e., removable denture and functional teeth) were entered in the second step (Model 2). After the addition of dental status-related variables in Model 2, 7.42% of the OHRQoL variance was explained, exhibiting an increase of 1.85% (Table 3). Oral hygiene variables (i.e., plaque index and tongue coating) were included in Model 3; no significant differences were found in the GOHAI scores. Adding oral hygiene variables increased the amount of OHRQoL variance by 0.75% (Table 3). Finally, in Model 4, variables related to oral function (i.e., xerostomia, dysphagia, occlusal condition, and masticatory performance) were added. Among participants, prefrailty (β = − 1.38, 95% CI: − 2.46, − 0.29), sarcopenia (β = − 2.59, 95% CI: − 4.78, − 0.40), xerostomia (β = − 3.93, 95% CI: − 5.36, − 2.50), dysphagia (β = − 2.78, 95% CI: − 4.08, − 1.48), and possession of fewer than 20 functional teeth (β = − 1.87, 95% CI: − 3.26, − 0.49) were significantly associated with reduced OHRQoL. Adding oral function related variables increased the amount of OHRQoL variance by 6.90%, and all variables combined explained 15.07% of the OHRQoL variance (Table 3).

**Table 2:** Hierarchical linear regression analysis of GOHAI scores associated with physical function, dental status, oral hygiene and oral function

	Model 1	Model 2	Model 3	Model 4
	β (95 % CI)	β (95 % CI)	β (95 % CI)	β (95 % CI)
Frailty				
Pre-frailty vs. robust	-2.23 (-3.27, -1.19)	-2.13 (-3.16, -1.10)	-2.08 (-3.18, -0.98)	-1.38 (-2.46, -0.29)
Frailty vs. robust	-2.90 (-6.47, 0.67)	-2.84 (-6.38, 0.70)	-3.17 (-6.89, 0.55)	-2.83 (-6.44, 0.79)
Sarcopenia	-3.88 (-5.92, -1.84)	-3.82 (-5.84, -1.79)	-3.42 (-5.66, -1.17)	-2.59 (-4.78, -0.40)
Removable denture (yes)	-	0.29 (-0.85, 1.43)	0.25 (-0.90, 1.40)	0.52 (-0.68, 1.72)
Functional teeth (<20 teeth)		-2.21 (-3.40, -1.03)	-2.39 (-3.65, -1.14)	-1.87 (-3.26, -0.49)
PI			0.20 (-0.65, 1.05)	0.18 (-0.65, 1.02)
Tongue coating			0.06 (-0.07, 0.20)	0.04 (-0.10, 0.17)
Xerostomia				-3.93 (-5.36, -2.50)
Dysphagia				-2.78 (-4.08, -1.48)
Occlusal condition				
B vs. A				-0.74 (-1.83, 0.34)
C vs. A				0.92 (-1.32, 3.16)
Masticatory				
Moderate vs. good				-0.70 (-1.64, 0.25)
Poor vs. good				-1.06 (-2.42, 0.03)

*PI* Plaque index

Model 1: This model included demographic factors, frailty and sarcopenia.

Model 2: This model included demographic factors, frailty, sarcopenia, removable denture and functional teeth.

Model 3: This model included demographic factors, frailty, sarcopenia, removable denture, functional teeth, plaque index and tongue coating.

Model 4: This model included demographic factors, frailty, sarcopenia, removable denture, functional teeth, plaque index, tongue coating, xerostomia, dysphagia, occlusal condition and masticatory performance.

**Table 3:** Summary of hierarchical linear regression models of GOHAI scores associated with physical function, dental status, oral hygiene and oral function

	Multiple R.	Adjust R^2^ (%)	R^2^ (%)	R^2^ change (%)	Sig. change	
Model 1	0.24	4.95	5.57	5.57	F change = 8.94	*p* <.001
Model 2	0.27	6.63	7.42	1.85	F change = 9.42	*p* <.001
Model 3	0.29	7.03	8.17	0.75	F change = 7.14	*p* <.001
Model 4	0.39	13.41	15.07	6.90	F change = 9.06	*p* <.001

Model 1: This model included demographic factors, frailty and sarcopenia.

Model 2: This model included demographic factors, frailty, sarcopenia, removable denture and functional teeth.

Model 3: This model included demographic factors, frailty, sarcopenia, removable denture, functional teeth, plaque index and tongue coating.

Model 4: This model included demographic factors, frailty, sarcopenia, removable denture, functional teeth, plaque index, tongue coating, xerostomia, dysphagia, occlusal condition and masticatory performance.

**Fig. 1:**
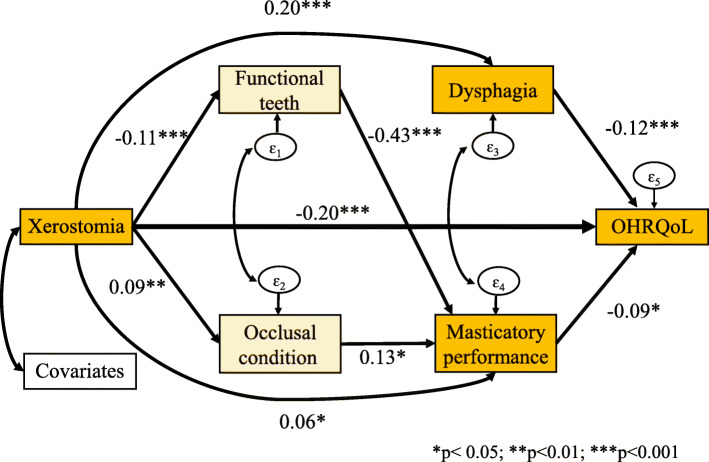
Path analysis framework

As displayed in Fig. 1, we hypothesized a pathway from xerostomia to OHRQoL through the mediators. The exogenous variables were xerostomia and covariates (i.e., age, sex, education level, frailty, and sarcopenia). The endogenous mediator variables were dental status, occlusal condition, dysphagia, and masticatory performance. Overall, the hypothesized model fit was good, with a goodness of fit of χ^2^ (1) =0.070, *p* = 0.791, RMSEA = 0.000, and CFI = 1.00. The results of the path analysis indicated that xerostomia (β_s_ = − 0.20) had the greatest overall effect on OHRQoL. Dysphagia had a significantly mediating effect on the association between xerostomia and OHRQoL (β_s_ = 0.20 and − 0.12, respectively, both *p* < .001). Moreover, masticatory performance exerted a significant mediating effect on the association between xerostomia and OHRQoL (βs = 0.06 and − 0.09, respectively, both *p* < .05). Potential mediating effects of the number of functional teeth (βs = − 0.11 and − 0.43, respectively, both *p* < .001) and occlusal condition (βs = 0.09 and 0.13, respectively; both *p* < .05) on the relationship between xerostomia and masticatory performance were also noted.

Table 4 presents the standardized path coefficients for the model. Xerostomia influenced OHRQoL both directly and indirectly and accounted for 13.6% of OHRQoL. The direct effect (β_s_ = − 0.20, *p* < .001) accounted for 83.3% of the total pathway; the indirect effect which combined two pathways, namely for dysphagia and masticatory performance (β_s_ = − 0.04, *p* < .001), accounted for 16.7% of the total pathway. Furthermore, xerostomia influenced masticatory performance both directly and indirectly and accounted for 38.2% of masticatory performance. The direct effect (βs = 0.06, *p* < .05) accounted for 50.0% of the total pathway, and the indirect effect, which combined two pathways, namely for the number of functional teeth and occlusal condition (β_s_ = 0.06, *p* < .001), accounted for 50.0% of the total pathway. However, the number of functional teeth and occlusal condition were found to exert no direct effects on OHRQoL through dysphagia.

**Table 4:** Standardized effects of physical function, dental status and oral function on GOHAI with correlated errors

Outcomes	Direct effect	Indirect effect	Total effect	R^2^ (%)
Functional teeth				15.1%
Xerostomia→ Functional teeth	-0.11***	−	-0.11***	
Occlusal condition				14.0%
Xerostomia→ Occlusal condition	0.09**	−	0.09**	
Dysphagia				9.2%
Functional teeth → Dysphagia	0.10	−	0.10	
Occlusal condition→ Dysphagia	0.11	−	0.11	
Xerostomia → Dysphagia	0.20***	-0.001	0.20***	
Masticatory performance				38.2%
Functional teeth→ Masticatory	-0.43***	−	-0.43***	
Occlusal condition→Masticatory	0.13*	−	0.13*	
Xerostomia→ Masticatory	0.06*	0.06***	0.12***	
GOHAI				13.6%
Functional teeth → GOHAI	0.03	0.03	0.06	
Occlusal condition → GOHAI	-0.03	-0.03*	-0.05	
Dysphagia → GOHAI	-0.12***	−	-0.12***	
Masticatory → GOHAI	-0.09*	−	-0.09*	
Xerostomia → GOHAI	-0.20***	-0.04***	-0.24***	

The covariates including age, gender and education

R^2^: the variance of endogenous variables that is explained

*p<0.05, **p<0.01, ***p<0.001

## Discussion

In contrast to studies using regression models to investigate the relationship between oral health status and quality of life [31], in this study, a pathway linking oral function to quality of life was examed and mediators in the model were identified. Testing a chain of relationships between an exogenous variable and an outcome enabled clarification of the transmission of one variable’s effects to another variable. Path analysis may also facilitate structuralize of the whole framework and establishment of various possible pathways, providing researchers and policy makers with various potential intervention strategies.

In this study, we found the strongest direct negative effect on OHRQoL to be exerted by xerostomia in older adults. According to the path model, dysphagia and masticatory performance may strongly mediate the effect of xerostomia on OHRQoL. This highlights a need to prevent xerostomia and reduce both its direct effects on quality of life and its indirect effects on older adults’ life satisfaction through dysphagia and masticatory performance. Eliminating the mediating effects of dysphagia and masticatory performance could prevent xerostomia from influencing quality of life. Moreover, in this study, mediating effects of the number of functional teeth and occlusal condition on the association between xerostomia and masticatory performance were observed. The direct effects of the number of functional teeth and occlusal condition on masticatory performance are also described in another study, which suggested that the risk of chewing difficulties is substantially increased in individuals with fewer pairs of opposing teeth [32].

Dysphagia was found to be the principal mediator of the effect of xerostomia on OHRQoL, and masticatory performance was identified as the secondary mediator. The indirect pathway of the effect of xerostomia through dysphagia may be attributable to the motion of swallowing, which is affected by the volume of saliva. Concordantly, swallowing may decrease during sleep because of the decrease in salivary secretions [33]. Overall, evidence strongly suggests that saliva is critical to both the frequency and efficiency of swallowing. Insufficient saliva may cause impaired swallowing, which can degrade the quality of life. A decline in oral functions could cause a negative self-perception in relation to oral health, as defined by the OHRQoL level. We demonstrate that the association between xerostomia and quality of life could be mediated by effect of swallowing function.

Our results indicate that masticatory performance is another potential mediator of the association between xerostomia and quality of life. People with xerostomia may have multiple oral and dental deficits, such as dental caries and periodontal disease, resulting in loss of teeth [34]. We used color-changing chewing gum to evaluate masticatory performance and discovered that older people with fewer than 20 functional teeth were more likely to have poor masticatory function. We also found potential mediating effects of the number of functional teeth and occlusal condition on the association between xerostomia and masticatory performance. The number of functional teeth and occlusal force were significantly related to objective masticatory function, which corroborates the findings of other research [35]. Several studies have also indicated that older people with fewer posterior natural teeth and fewer teeth overall are more likely to experience difficulty chewing [36]. The number of residual teeth is clearly paramount to mastication. A study conducted in Japan demonstrated that the retention of more than 20 teeth is necessary for effective masticatory ability [37]. It is reasonable to suppose that low masticatory ability in older people caused by the loss of multiple teeth affects dietary choices and therefore reduces quality of life. As a result of impaired masticatory ability, older people are unable to eat according to their preferences and have particularly low intake of meat, fruits and vegetables. Therefore, masticatory ability is vital for life satisfaction among the older population.

The results of the moderated mediation analysis indicate that dysphagia represents a more prominent pathway among individuals with xerostomia than among those without xerostomia. Frequent sipping water, chewing sugar-free gum or sucking on sugar-free candy, and avoiding caffeine, tobacco, alcohol and dry or difficult-to-chew foods may relieve xerostomia symptoms [38]. In addition, massaging the three paired major salivary glands (parotid, submandibular, and sublingual) for 10 min, three times daily before each meal may mitigate the subjective sensation of thirst. These measures for reducing symptoms associated with xerostomia may help older people to swallow more easily during meals, which may improve their OHRQoL. Moreover, oral exercises (i.e., neck exercises, salivary gland massages, and vocal exercises) may reduce the direct and indirect effects of xerostomia on quality of life. Oral exercise effectively improves the oral diadochokinesis rate and reduces xerostomia, dysphagia, and poor masticatory performance [38].

The number of teeth is a crucial factor affecting masticatory function. However, the tongue is also vital for mastication and determines older people’s ability to mix food because the tip of the tongue plays a key role in masticatory movement. Therefore, increased tongue movement in older people may compensate for decreased mastication. Furthermore, a simple examination that uses oral diadochokinesis to identify the preclinical signs of dysphagia could counter dysphagia and reduced quality of life in community-dwelling older people. An oral diadochokinesis test, in which three syllables are rapidly repeated six times, can be used for straightforward diagnosis of hypofunctional oral diadochokinesis.

Some possible limitations of this study should be noted. First, a cross-sectional design adopted restricts interpretation of the causal processes underlying oral health outcomes; thus, possible reverse causality cannot be ruled out. Second, the used of a questionnaire to evaluate the dryness of the mouth is not considered an objective method; however, xerostomia is just the subjective sensation of dryness of mouth, and is not necessarily associated with reduced salivary flow. Although path analysis was used in the present study, direct and indirect causal relations between xerostomia and impaired OHRQoL could not be determined. Moreover, existence or non-existence of dysphagia was evaluated based on the self-reported difficulties of swallowing, which may jeopardize the causal relations among xerostomia, dysphagia and OHRQoL. In the future, researchers should use a more objective method for evaluating oral function, such as saliva flow rate measurement and a videofluoroscopic swallowing test, which is considered a more rigorous means of revealing the associations of factors to oral health status. Data collection with objective measures in multiple community-based settings is relatively inconvenient, time consuming, and expensive for a large-scale survey; a questionnaire is a more feasible and economic option. Studies have validated that self-reporting scales for dry mouth and dysphagia provide high validity and reliability [22, 39]. Third, because of language barriers, interviewers had to switch between Mandarin and Taiwanese, which may have caused interviewer bias. However, interviewers were trained before the survey to minimize this bias. Fourth, we did not evaluate tongue pressure, which is related to swallowing motion, among the older people in this study. Fifth, social and cultural variables were not considered in our model because of population variations in social, economic, cultural, and technological characteristics that could be associated with physical conditions. Oral health problems such as dysphagia may be prevalent in some subpopulations of society, and this may lead to dehydration, malnutrition, or even early mortality. Further studies are required to identify potential mediators of the relationship by which oral health factors influence quality of life in a broader framework. Finally, older adults were recrutied in Kaohsiung City, representing only a subgroup that can not necessarily be considered representative of the rest of the population.

## Conclusions

Our findings suggest that the mediating roles of dysphagia and masticatory performance in the association between xerostomia and OHRQoL were significant and deserve further attention. A community-based educational program targeting oral function improvements in older people may be a promising strategy for improving OHRQoL. In addition, our results suggest that early screening for swallowing and masticatory function is essential to prevent or delay the onset of complications. Government and health agencies should provide older adults in community settings with oral health services, including regular dental inspections and screening of oral function (i.e. xerostomia, chewing and swallowing). Referral to oral health specialists is also recommended for older adults to improve bite strength and the number of functional teeth.

## Data Availability

The datasets generated and/or analyzed during the current study are not publicly available due to maintain participant privacy and confidentiality requirements but are available from the corresponding author on reasonable request.

## Abbreviations

ADL: Activities of Daily Living
CFI: Comparative Fit Index
GDS: Geriatric Depression Scale
GOHAI: Geriatric Oral Health Assessment Index
OHRQoL: Oral health-related quality of life
RMSEA: Root Mean Square Error of Approximation
SARC-F: Strength, assistance in walking, rising from a chair, climbing stairs, and falls
SEM: Structural equation model
SOF: Study of Osteoporotic Fractures Index
SPMSQ: Short portable mental status questionnaire
VIF: Variance inflation factor

## References

[1] Agostini BA, Cericato GO, Silveira ERD, Nascimento GG, Costa FDS, Thomson WM (2018). How common is dry mouth? Systematic review and meta-regression analysis of prevalence estimates. Braz Dent J.

[2] Locker D (1993). Subjective reports of oral dryness in an older adult population. Community Dent Oral Epidemiol.

[3] Cassolato SF, Turnbull RS (2003). Xerostomia: clinical aspects and treatment. Gerodontology..

[4] Genkai S, Kikutani T, Suzuki R, Tamura F, Yamashita Y, Yoshida M (2015). Loss of occlusal support affects the decline in activities of daily living in elderly people receiving home care. J Prosthodontic Res.

[5] Baijens LW, Clavé P, Cras P, Ekberg O, Forster A, Kolb GF (2016). European Society for Swallowing Disorders - European Union geriatric medicine society white paper: oropharyngeal dysphagia as a geriatric syndrome. Clin Interv Aging.

[6] García-Peris P, Parón L, Velasco C, de la Cuerda C, Camblor M, Bretón I (2007). Long-term prevalence of oropharyngeal dysphagia in head and neck cancer patients: impact on quality of life. Clinical nutrition (Edinburgh, Scotland).

[7] Takata Y, Ansai T, Awano S, Fukuhara M, Sonoki K, Wakisaka M (2006). Chewing ability and quality of life in an 80-year-old population. J Oral Rehabil.

[8] Bayram H, Ilgaz F, Serel Arslan S, Demir N, Rakıcıoğlu N. The relationship between dysphagia, oral health, masticatory performance and activities of daily living in elderly individuals as assessed by the eating assessment tool. Prog Nutr. 2020;23.

[9] Furuta M, Yamashita Y (2013). Oral health and swallowing problems. Curr Phys Med Rehab Rep.

[10] Liang YH, Chou C, Chen YJ, Chou YF, Lin CY, Chou C (2020). Impact of periodontal disease and chewing ability on the quality of life of the elderly in an affluent community. J Formos Med Assoc.

[11] Rebelo MA, Cardoso EM, Robinson PG, Vettore MV (2016). Demographics, social position, dental status and oral health-related quality of life in community-dwelling older adults. Qual Life Res.

[12] Enoki K, Ikebe K, Matsuda KI, Yoshida M, Maeda Y, Thomson WM (2013). Determinants of change in oral health-related quality of life over 7 years among older Japanese. J Oral Rehabil.

[13] Moriya S, Tei K, Murata A, Muramatsu M, Inoue N, Miura H (2012). Relationships between geriatric Oral health assessment index scores and general physical status in community-dwelling older adults. Gerodontology..

[14] Newton JT, Bower EJ (2005). The social determinants of oral health: new approaches to conceptualizing and researching complex causal networks. Community Dent Oral Epidemiol.

[15] Pfeiffer E (1975). A short portable mental status questionnaire for the assessment of organic brain deficit in elderly patients. J Am Geriatr Soc.

[16] Liu YM, Chang HJ, Wang RH, Yang LK, Lu KC, Hou YC (2018). Role of resilience and social support in alleviating depression in patients receiving maintenance hemodialysis. Ther Clin Risk Manag.

[17] Liu CY, Lu CH, Yu S, Yang YY (1998). Correlations between scores on Chinese versions of long and short forms of the geriatric depression scale among elderly Chinese. Psychol Rep.

[18] Kline RB (2015). Principles and practice of structural equation modeling.

[19] Zhou YC (2010). Validation of the geriatric Oral health assessment index in Taiwan: Kaohsiung Medical University.

[20] Thomson WM, van der Putten GJ, de Baat C, Ikebe K, Matsuda K, Enoki K (2011). Shortening the xerostomia inventory. Oral Surg Oral Med Oral Pathol Oral Radiol Endod.

[21] Hahnel S, Schwarz S, Zeman F, Schafer L, Behr M (2014). Prevalence of xerostomia and hyposalivation and their association with quality of life in elderly patients in dependence on dental status and prosthetic rehabilitation: a pilot study. J Dent.

[22] Papadopoulou SL, Exarchakos G, Christodoulou D, Theodorou S, Beris A, Ploumis A (2017). Adaptation and assessment of reliability and validity of the Greek version of the Ohkuma questionnaire for dysphagia screening. Int Arch Otorhinolaryngol.

[23] Wada S, Kawate N, Mizuma M (2017). What type of food can older adults masticate?: evaluation of mastication performance using color-changeable chewing gum. Dysphagia..

[24] Ikebe K, Matsuda K, Murai S, Maeda Y, Nokubi T (2010). Validation of the Eichner index in relation to occlusal force and masticatory performance. Int J Prosthodont.

[25] Winkel EG, Roldan S, Van Winkelhoff AJ, Herrera D, Sanz M (2003). Clinical effects of a new mouthrinse containing chlorhexidine, cetylpyridinium chloride and zinc-lactate on oral halitosis. A dual-center, double-blind placebo-controlled study. J Clin Periodontol.

[26] Ensrud KE, Ewing SK, Cawthon PM, Fink HA, Taylor BC, Cauley JA (2009). A comparison of frailty indexes for the prediction of falls, disability, fractures, and mortality in older men. J Am Geriatr Soc.

[27] Woo J, Leung J, Morley JE (2014). Validating the SARC-F: a suitable community screening tool for sarcopenia?. J Am Med Dir Assoc.

[28] Fueki K, Yoshida E, Igarashi Y (2011). A structural equation model relating objective and subjective masticatory function and oral health-related quality of life in patients with removable partial dentures. J Oral Rehabil.

[29] Furuta M, Komiya-Nonaka M, Akifusa S, Shimazaki Y, Adachi M, Kinoshita T (2013). Interrelationship of oral health status, swallowing function, nutritional status, and cognitive ability with activities of daily living in Japanese elderly people receiving home care services due to physical disabilities. Community Dent Oral Epidemiol.

[30] Buco C, Buenviaje KAC, Bulan RBC, Cabana RJL, Cabuhat MKS, Bongar MVV (2018). Developing and testing a model of quality of life among chronically-ill, community-dwelling older adults: a structural equation model. Arch Gerontol Geriatr.

[31] Inukai M, John MT, Igarashi Y, Baba K (2010). Association between perceived chewing ability and oral health-related quality of life in partially dentate patients. Health Qual Life Outcomes.

[32] Hatch JP, Shinkai RS, Sakai S, Rugh JD, Paunovich ED (2001). Determinants of masticatory performance in dentate adults. Arch Oral Biol.

[33] Lear CS, Flanagan JB, Moorrees CF (1965). The frequency of deglutition in man. Arch Oral Biol.

[34] Villa A, Connell CL, Abati S (2015). Diagnosis and management of xerostomia and hyposalivation. Ther Clin Risk Manag.

[35] Takagi D, Watanabe Y, Edahiro A, Ohara Y, Murakami M, Murakami K (2017). Factors affecting masticatory function of community-dwelling older people: investigation of the differences in the relevant factors for subjective and objective assessment. Gerodontology..

[36] Ueno M, Yanagisawa T, Shinada K, Ohara S, Kawaguchi Y (2008). Masticatory ability and functional tooth units in Japanese adults. J Oral Rehabil.

[37] Manabe K, Tanji F, Tomata Y, Zhang S, Tsuji I (2019). Preventive effect of Oral self-care on pneumonia death among the elderly with tooth loss: the Ohsaki cohort 2006 study. Tohoku J Exp Med.

[38] Sugiyama T, Ohkubo M, Honda Y, Tasaka A, Nagasawa K, Ishida R (2013). Effect of swallowing exercises in independent elderly. Bull Tokyo Dental College.

[39] Thomson WM, Chalmers JM, Spencer AJ, Williams SM (1999). The Xerostomia inventory: a multi-item approach to measuring dry mouth. Community Dent Health.

